# Is It Necessary Managing Carnivores to Reverse the Decline of Endangered Prey Species? Insights from a Removal Experiment of Mesocarnivores to Benefit Demographic Parameters of the Pyrenean Capercaillie

**DOI:** 10.1371/journal.pone.0139837

**Published:** 2015-10-21

**Authors:** Rubén Moreno-Opo, Iván Afonso, José Jiménez, Mariana Fernández-Olalla, Jordi Canut, Diego García-Ferré, Josep Piqué, Francisco García, Job Roig, Jaime Muñoz-Igualada, Luis Mariano González, José Vicente López-Bao

**Affiliations:** 1 Ministry of Agriculture, Food and Environment, Madrid, Spain; 2 TRAGSATEC, Madrid, Spain; 3 MUSIA, La Plana, Lleida, Spain; 4 Association for the Conservation of Capercaillie (ACU), Buseu, Baix Pallars, Spain; 5 Institute of Research in Game Resources-CSIC, Ciudad Real, Spain; 6 ETSI de Montes. University Politécnica Madrid, Madrid, Spain; 7 Alt Pirineu Natural Park, Llavorsí, Spain; 8 Departament d´Agricultura, Ramaderia, Pesca, Alimentació i Medi Ambient, Generalitat de Catalunya, Barcelona, Spain; 9 Research Unit of Biodiversity, Oviedo University, Mieres, Spain; 10 Grimsö Wildlife Research Station, Swedish University of Agricultural Sciences, Riddarhyttan, Sweden; University of Lleida, SPAIN

## Abstract

Mesopredator control has long been used to alleviate the effect of elevated predation pressure on vulnerable, threatened or valuable species. However, the convenience of using mesopredator controls is technically questionable and scientifically-sound research is therefore required to evaluate the impact of predation on prey case by case. In this study we evaluated the effect of the alteration of terrestrial mesopredator dynamics on the demographic parameters of a relict capercaillie *Tetrao urogallus aquitanicus* population currently in decline for which the impact of predation has not previously been assessed. We used a six-year mesocarnivore removal experiment (2008–2013) together with seven-years of previous demographic information on capercaillies (1999–2007) within a before-after control-impact (BACI) design to evaluate the effect of mesocarnivore removal on capercaillie demographic parameters and on spatial behaviour of the most frequent predatory mesocarnivores of the capercaillie (*Martes* spp. and red fox *Vulpes vulpes*). Using a dynamic site-occupancy approach, the reduction of mesocarnivore population levels as a result of removal was clear for marten species, mainly during key months for capercaillie reproduction, but not for the red fox. Our results show that the breeding success of capercaillies was enhanced in areas where carnivores were removed and was inversely related to the occupation level of the studied mesocarnivores, although being only significant for *Martes* spp. Moreover, capercaillie predation rates were lower and adult survival seemingly higher in treatment during the removal phase. Cost-effective, long-term management interventions to ensure the recovery of this threatened capercaillie population are discussed in the light of the results. At our study area, the decision for implementing predation management should be included within a broader long-term conservation perspective. In this regard, a more feasible and sustainable management intervention in ecological and economic terms may be to balance the impact of mesocarnivores on capercaillies through the recovery of apex predators.

## Introduction

There is growing recognition of the important role that apex predators play in ecosystems (i.e. top-down effects and trophic cascades) [1–4], as well as the complexity of the ecosystem processes in which they are involved. For example, top-down impacts of apex predators are mediated by ecosystem productivity and depend on predator densities [5–7]. Apex predators control mesopredators [3,6] and their loss leads to *mesopredator release* translating, for instance, into increased predation pressure on vulnerable prey species [1,6]. The presence or absence of apex predators, and changes in their abundance, leads to shifts in the occupancy and abundance of mesopredators, especially those not specialized in the exploitation of a particular resource [1,8]. As a consequence, the demographic effects of predation on prey populations are closely related to the ecological interactions between predators [9–13].

The absence of apex predators has multiple consequences on prey populations and ecosystems, biodiversity conservation, wildlife management and even the economy [6,14–16]. Management interventions such as mesopredator controls have long been used to alleviate the effect of elevated predation pressure–in part due to the absence of apex predators-on vulnerable, threatened or valuable species [17–20]. Several systematic reviews have shown that mesopredator control may be an effective strategy to increase hatching and fledging success, as well as post-breeding population numbers in several avian prey species, especially ground-nesting birds [18,21,22]. However, the influence of this intervention on other prey population parameters such as recruitment and survival or its effectiveness over time is far less clear [21]. On the other hand, this intervention is highly controversial, is financially and logistically expensive, and time-consuming [21,23]. Finally, it often has a small effect on predator populations [21,24], although evaluations are rarely done [19,21,25]. Consequently, when conserving vulnerable prey species, the convenience of using mesopredator controls is questionable and scientifically-sound research is therefore required to evaluate the impact of predation on prey populations, the effectiveness and suitability of mesopredator controls and, on the other hand, to explore the feasibility of using alternative interventions, such as restoring the predator community, delineating effective future management practices.

Perturbation experiments [26] are generally used to generate evidence to identify limited or regulated prey populations (i.e., experiments including the temporal or permanent removal of predators) [27,28], but they can also be useful to elucidate the potential impact of the reverse, namely the mesopredator release process. This manipulative experimentation can provide conservation practitioners with useful evidence to guide informed decisions in future conservation planning under an adaptive management approach [27]. In perturbation experiments aimed at understanding the impact of mesopredators on prey populations, mesopredators must be removed from treatment areas or their numbers reduced significantly. This would allow the comparison of the effects of reduced abundance of mesopredators on prey population parameters with unaffected control sites and, preferably, with their values before the beginning of the experiment (before-after control-impact design, BACI). Similarly, it is important to assess the effects that this type of experiments have on the populations of the species to be removed, to apply best-evidenced conservation and management measures [19,26].

Capercaillie *(Tetrao urogallus)* populations are declining in most of their range and are especially vulnerable in southern Europe [29,30]. Several non-mutually exclusive factors have been proposed as the main causes of capercaillie decline, including land use change, habitat fragmentation and degradation, climate change, human disturbance, competition with ungulates, or predation [29]. However, the strength of the impact of each factor contributing to the decline and their synergies are unclear, and therefore, decision-making processes are frequently based on poor evidence. High predation levels have been identified as a potential factor limiting capercaille populations, and previous experience has suggested that predator control may have a positive effect on capercaillie breeding success [31–33]. However, although most studies have evaluated the effect of mesopredator removal in terms of improved breeding success [31–33], less attention has been paid to changes in predation rate or adult survival rate, which are influential parameters in capercaillie population growth rate [30]. On the other hand, its effects seem to be mainly short-term [34] and the impact of this intervention on mesopredators is poorly understood [19,35].

In this study, we aimed to understand the impact of a mesopredator removal intervention on both capercaillies and predator populations, in a process consistent with the principles and conditions for adaptive management (i.e. complex and changing systems, need for urgent action, incomplete information on ecology of the target species and expectancy of improvement and learning on the issue) [36]. We analyzed the effect of a six-year removal experiment of terrestrial mesocarnivores to answer two main questions: i) what is the impact of mesopredator removal on predator occupation? and ii) what is the effect of this intervention on capercaillie breeding success, adult survival and predation rates? Our final goal was to provide a discussion on the effect of predation pressure on this endangered population and delineate the best associated management options for reducing the threat level of the vulnerable Pyrenean capercaillie populations, including the discussion of the role of the reintroduction of locally extinct apex predators in the area.

## Material and Methods

### The studied Pyrenean capercaillie population and area

Capercaillies have experienced population declines and local extinctions all over western and central Europe in recent decades [29]. Together with the Cantabrian one, the Pyrenean population is genetically isolated from other European populations [29] and has been considered as an evolutionary significant unit [37]. This population has suffered a decrease of 31% in the number of males since the early 1990s [38] and consequently is classified as vulnerable under Spanish law [39]. No remarkable changes in habitat quality (i.e. fragmentation, forest cover) have been observed in this area that could explain the observed decline in the population. On the contrary, forested areas have significantly increased both in surface area and succesional stage during the last 50 years across the Pyrenean region, since traditional land uses carried out during the nineteenth and twentieth centuries (e.g. logging, grazing, agriculture) have mostly been abandoned [40,41]. Nevertheless, ungulates appear to have increased in population size while apex predators declined significantly during the last century [42].

**Fig 1:**
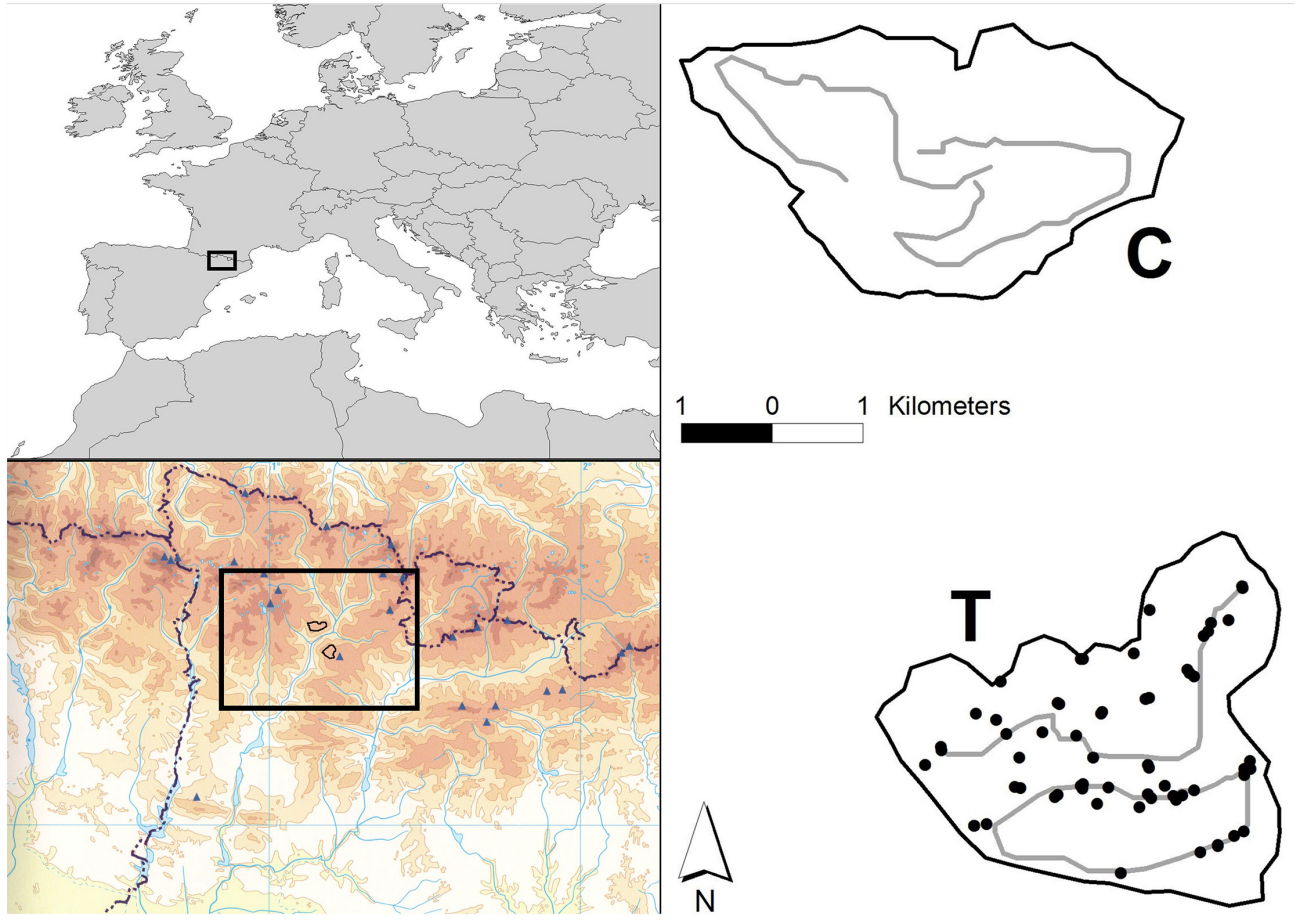
Study area of the mesocarnivore removal experiment performed to evaluate the effect on population parameters of the vulnerable Pyrenean capercaillie *Tetrao urogallus aquitanicus*, within the western Palearctic region (left, above) and the Pyrenees Mountain range (left, below, source: Catalan Geographic Institute http://www.icgc.cat/). The two study plots (control = C, and treatment = C) scaled, the foot transects for mesocarnivore monitoring (in grey) and the locations of removed mesocarnivores (black dots in T plots) are also shown.

The study area is located in the Alt Pirineu Natural Park (Catalonia, 42°27´33.17´´N 1°10´1.67´´E), which includes the highest number of leks of the Pyrenean capercaillie in Spain (187 leks, 512–573 males, Fig 1) [38]. The studied population mainly inhabits forests dominated by mountain pine *Pinus uncinata* with a bilberry *Vaccinium myrtillus* and rhododendron *Rhododendron ferrugineum* understory located between 1500 and 2300 m above sea level (a.s.l.) Native apex predators are extinct in the area (i.e. Eurasian lynx *Lynx lynx* and grey wolf *Canis lupus*) or very scarce and peripheral to the selected study plots (brown bear *Ursus arctos*, Fig 1) [42].

### The mesopredator removal experimental design

We carried out a multi-species mesopredator removal experiment to avoid the counterbalance of the reduction in prey losses by one mesocarnivore with predation by others [43,44]. We then focused on generalist terrestrial mesocarnivores that could potentially predate on, at least, one stage of the capercaillie reproductive cycle [44–46]. Thus, our target species included the pine and stone marten *Martes martes* and *Martes foina*, respectively, red fox *Vulpes vulpes*, wildcat *Felis sylvestris*, Eurasian badger *Meles meles* and common genet *Genetta genetta*.

From 2008 to 2013 we carried out a mesocarnivore removal experiment in Alt Pirineu Natural Park. We first selected two study plots (Fig 1) as isolated forest blocks with similar attributes in: i) size (971 vs. 1009 ha), ii) habitat characteristics (91.3 and 93.8% of forest cover in each area), with no significant changes in forest cover during the study period as well as no timber exploitation or forest management, iii) number of leks (3 leks – 12 males–vs. 3 leks – 15 males-), and iv) number of estimated non-juvenile population ranging from 30 to 40 individuals in each plot. Study plots were selected at a distance sufficient to minimize the probability that the same individual carnivore would overlap the two study plots. Therefore, considering the home range size of different radiotagged (Very High Frequency, VHF) mesopredators in the area (based on pine and stone martens and using the minimum convex polygon with 100% of positions; stone marten: 262.87 ± 124.96 ha, n = 6; pine marten: 155.84 ± 38.78 ha, n = 2, authors´ unpublished data) and the landscape configuration (different valleys in a highly rugged landscape separated by rivers), we considered that an Euclidean distance of 5 km was sufficient to consider the two plots as independent [47,48]. In fact, from a total number of eight marten individuals captured and radiotagged, we did not record a single location from the same individual in both plots.

We then randomly assigned the category of treatment (hereafter T) or control (hereafter C) to each plot. The removal of mesocarnivores was carried out only in T. Mesocarnivore removals were performed annually between 2008 and 2013, with the annual effort being homogeneous over time (Spearman rank correlation, r_s_ = -0.25; P = 0.625, for total night-traps, Table 1; see Fig 1 for the spatial distribution of traps). Removals were focused before and during capercaillie laying (10^th^ May to 15^th^ July), hatching (5^th^ June to 5^th^ August) and/or rearing periods (15^th^ June to 30^th^ August, Table 1). Carnivores were live-trapped using several methods: self-made box-traps, Tomahawk box-traps, Belisle and Collarum [49] (Table 1). Traps were baited with live-prey (domestic pigeons) or with several types of odorous lures and pieces of meat, and checked daily early in the morning and through automatic alerts using GPS-GPRS transmitters. All captured carnivores were translocated to approximately 100 km from T, with the exception of red foxes, which were legally euthanized (n = 12) in compliance with its legal status as hunting species in Spain. To check for potential *homing* effects [50], which would invalidate our experiment, we marked a random sample of every translocated species (about one in every three captures sequentially) using subcutaneous tags (Freevision, 0.15 g, ISO 11784/785), to determine if they were re-captured after translocations in T. We used a simple 2 x 2 BACI experimental design, in which parameters of interest were measured before and after treatment, for both control and treatment conditions, because of the logistical constraints of this type of large-scale experiments and the spatial behaviour of target species (plots of ca. 1,000 ha and mesocarnivores with home ranges of several hundreds of ha).

**Table 1:** Removal effort of mesocarnivores in treatment plot (T). Months in which removal was applied, the number of different traps installed and the number of nights during which traps operated (night-traps) are shown. The efficiency of the removal was calculated as the number of captures of targeted mesocarnivores divided by the number of night-traps*100. The same data are provided for each of the four different types of traps: live-baited traps (cage traps baited with live feral pigeons *Columba livia* var. dom.), cage traps for martens (baited with several types of dead baits), belisle traps (http://belisletrap.com/) and collarum traps (http://collarum.com/).

Year	Period of captures	Total installed traps (n)	Total night-traps (n)	Captures/night-traps	Live-baited traps installed (n)	Captures in live-baited traps/night-traps	Cage traps for marten installed (n)	Captures in cage traps for marten/night-traps	Belisle installed (n)	Captures in belisle /night-traps	Collarum installed (n)	Captures in collarum /night-traps
2008	February-June	76	4660	0.47	12	1.36	43	0.15	10	0.45	11	0.52
2009	January-June	57	5221	0.44	11	0.75	35	0.33	6	0	5	0
2009–2010	November-June	93	12703	0.11	11	0.06	45	0.17	8	0	4	0
2010–2011	December-June	68	4716	0.17	5	0.52	42	0.16	10	0	0	
2012	June-August	40	2947	0.27	0		30	0.31	10	0.14	0	
2013	January-August	45	4294	0.16	0		30	0.19	10	0	0	0

### Mesocarnivore monitoring

In both T and C plots, we monitored the occurrence and abundance of mesocarnivores over time based on sign counts. To do this, we searched for faeces and footprints from each mesocarnivore species along fixed transects situated on forest roads or trails, avoiding misidentification between martens and red fox as much as possible [51] (Fig 1). A similar sampling effort (transect length) was invested per plot (14.4 and 14.8 km, in T anc C, respectively) (Fig 1). Two trained observers performed the surveys by foot on a monthly basis, and removed all signs once recorded. For each sampling period, the presence of each species was established according to the presence of signs regardless of their numbers. Although we removed individuals from the entire mesocarnivore community and recorded the presence of all species during transects, it is worth noting that we focused on red fox and marten species as a surrogate of the whole mesocarnivore community since the rest of the targeted mesocarnivores were insufficiently detectable with this procedure or were not sufficiently abundant [52], limiting subsequent analysis. The protocol did not allow us to distinguish between pine and stone martens, in the absence of DNA analysis [53]; hence, we considered these species jointly as *Martes* spp. for subsequent analysis. To avoid spatial correlation between closely situated transects and in light of the home-foraging ranges of red fox and marten species (about 100 to 250 ha) ([48,54,55], authors´ unpublished data) alternative stretches of 200 m in each transect and a distance between stretches of 2,000 m were considered.

### Capercaillie monitoring

Every year from 1999 to 2013, we carried out route censuses [56] to collect data on capercaillies in C and T plots. Route censuses consisted of a line transect by 8–20 people arranged at 10–20 m intervals walking simultaneously through forest patches within the T and C plots. At the time of the census during the first to third week of August chicks were well developed and able to fly [57]. The numbers of flushed females and fledglings were recorded, as well as the area covered.

For T and C, we estimated breeding success, adult survival and predation rates for capercaillies. We considered breeding success as the number of fledglings per hen. To evaluate changes in the survival rate of capercaillies we monitored adult birds (i.e., from their second calendar year) both in T and C during the experiment. From 2008 to 2013, we used fishing nets placed at leks during the display period (May) for trapping adult males. On the other hand, lure sounds and fishing nets during the chick-rearing period (June to August) were perfomed for capturing females. Once captured, adult capercaillies were fitted with VHF transmitters attached as collars (Holohill Systems Ltd. and Biotrack Ltd.; S1 Fig) [58]. Handling between capture and release lasted less than 3 minutes per individual. Biometrics and biological samples were not taken to avoid increasing stress levels [59]. Subsequently, we monitored the survival of capercaillies by recording the geographic location of all individuals and their status (alive or dead) on a biweekly basis. We only considered animals with a minimum effective monitoring time of 30 days for subsequent analyses (n = 33). Eleven capercaillies were captured in plot T (6 males and 5 females) and 22 in plot C (13 males and 9 females).

Finally, we estimated mesocarnivore predation rates in T and C plots using dead capercaillies, including whole carcasses, remains and feather concentrations found during surveys. The number of casualties was considered as a surrogate of predation rate by mesocarnivores in each studied plot and year. We only included in the analyses casualties derived from active predation events, also discarding those in which the species causing the predation could be misidentified due to secondary scavenging [60]. We assigned mammals as causing the predation according to visual evidence in the form of biting, eating, displacing, hiding and/or situating the prey (S2 Fig) [61]. Unknown cases (other than predation by raptors) were also recorded.

### Data analysis

We evaluated the impact of our mesopredator removal experiment on mesopredators by quantifying changes in the occupancy patterns over time focusing on red fox and *Martes* spp. To derive levels of occupancy by these species in T and C, data on sign counts were grouped into seasons, considering the first three quarters of the year (January to August), coinciding with the removal period. We did not include the rest of the year (September-December) due to the reoccupation of T by dispersing mesocarnivores which replaced removed individuals and reduced divergences in occupancy levels [62]. As the trends of relative abundances of mesopredators are difficult to quantify directly due to their inacurate detectability [63], we used a dynamic site-occupancy model approach to assess temporal variation in *Martes* spp. and red fox occupancy in both plots. For each plot, and red fox and *Martes* spp. species separately, we built a hierarchical Bayesian model that simultaneously evaluated an ecological submodel (presence-absence) linked to an observation submodel (detection-non-detection derived from counting signs grouped into seasons, averaging three consecutive months) and extended to the whole study period [64,65]. To calculate the site-occupancy values we used the dynamic occupation code from Kéry and Schaub [66]. For all models, we ran 3 chains of the MCMC sampler with 250 000 iterations each, discarding 10 000 iterations as burn-in using the software JAGS [67] and R 3.1.1 [68]. To check for chain convergence, we calculated the Gelman-Rubin statistic R-hat [69]. Values below 1.1 indicated convergence. In our results, all model parameters had R-hat <1.1, and we reported the trends in seasonal occupation *ψ*–adjusted to the probability of detection *p—*of both plots and 95% Bayesian credible intervals (95BCI) for all parameters.

To test for the existence of effects of the mesocarnivore removal experiment on capercaillie breeding success, we evaluated differences in breeding success between T and C following a BACI experimental design [70]. Thus, we fitted a zero inflated GLMM with Poisson distribution errors and log link function [71] to evaluate how the treatment and period (interaction term: treatment x period) influenced breeding success, i.e. the number of fledglings per female (count data). We compared the fit of this zero-inflated model structure against a Poisson and a Negative Binomial model strucure using the Akaike Information Criterion (AIC) [72]. Although this forest landscape did not change remarkably during the study period, we included in the model two additional climate factors that may have influenced the probability of observing a female with fledglings (i.e. potential impact on chick survival) [73,74] in order to statistically control their potentially confounding effect. Thus, we associated to each female record a measure of rainfall (mm) and the minimum temperature (°C) during the incubation and first phase of the chick-rearing period (May and June). In addition, because the probability of detecting females relied on the size of the census area, we decided to include the yearly sampling effort (ha) as a covariate in the model to control for heterogeneity in sampling effort. Year was fitted as a random effect in the model to account for year effects. Additionally, to deepen our understanding of the effect of the predator-capercaillie relationship, we performed a correlation analysis between the breeding success values of capercaillie and the site-occupancy results of *Martes* spp. and red fox during the breeding period of capercaillie (second and third quarter of the year) and both in T and C plots in each year of study.

For each plot, we computed the adult survival rate of VHF-marked capercaillies using the Kaplan-Meier product-limit method and tested for differences between T and C cumulative survival values [75] using the package *“survival”* for R [76]. Finally, every year in both C and T plots, considering the sampling effort (ha) and the number of predation events of capercaillies found, we calculated a predation rate by sampling unit (ha) by mesocarnivores on capercillies. We then built a GLMM with Gaussian error distribution and identity link to explore whether predation rate differed between C and T plots following a BACI experimental design (interaction term: treatment x period). Sampling effort was included as a covariate in the model, since this factor affects the probability of finding a predation event, and year was included as a random factor. We used the “*glmmADMB*” package [77] to run the GLMMs and the ‘‘*car*” package for R to calculate and Wald *χ2* to evaluate the significance levels for model parameters [78].

### Ethics statement

This study was carried out on public lands in strict compliance with the European (Directives 92/43/CEE and 147/2009/CE) and Spanish (Act 42/2007) legislation on the protection of threatened wildlife. Exceptional permits for trapping, movement and equipping the target species with transmitters–all of them protected under the Spanish law—were obtained from the competent authorities (permits number: SF/008/2008; Departament de Medi Ambient i Habitatge and Alt Pirineu Natural Park of Generalitat de Catalunya). The protocols used were consistent with best practices and technical and scientific recommendations related to animal welfare and efficiency.

## Results

### Effect of mesocarnivore removal on predators

A total of 67 mesocarnivores were translocated and 12 foxes were euthaniasized between 2008 and 2013 in the T plot (an average of 11.2 and 2.0 individuals per year, respectively; Table 2). We captured individuals from all the mesocarnivore species considered and observed a negative trend over time in the number of captures (Spearman rank correlation, r_s_ = -0.905; *P* = 0.013; Table 2). In general, the number of mesocarnivores trapped was greater during the first 2–3 years of the study diminishing over time (Table 2). However, the observed patterns in the number of mesocarnivores trapped was not due to changes in trapping effort, either, evaluated as the number of captures *vs*. the number of total traps installed (F_1,5_ = 0.726; *P* = 0.442, Table 1) or as the number of captures *vs*. the number of total trap-nights (F_1,5_ = 0.098; *P* = 0.768; Table 1). No tagged and translocated individuals were captured again in the T plot.

**Table 2:** Number of individuals of the different targeted mesocarnivore species captured during the removal experiment in the treatment plot (T) during the six-year fieldwork period.

					Year			
Species		2008	2009	2009–2010	2010–2011	2012	2013	Total captures (n)
Stone marten	*Martes foina*	2	10	9	4	5	3	33
Pine marten	*Martes martes*	1	1	0	1	0	3	6
Red fox	*Vulpes vulpes*	6	2	1	1	1	1	12
Eurasian badger	*Meles meles*	3	1	2	0	0	1	7
Wildcat	*Felis silvestris*	2	3	1	1	0	0	7
Common genet	*Genetta genetta*	7	4	1	0	2	0	14
	Total	21	21	14	7	8	8	79

**Fig 2:**
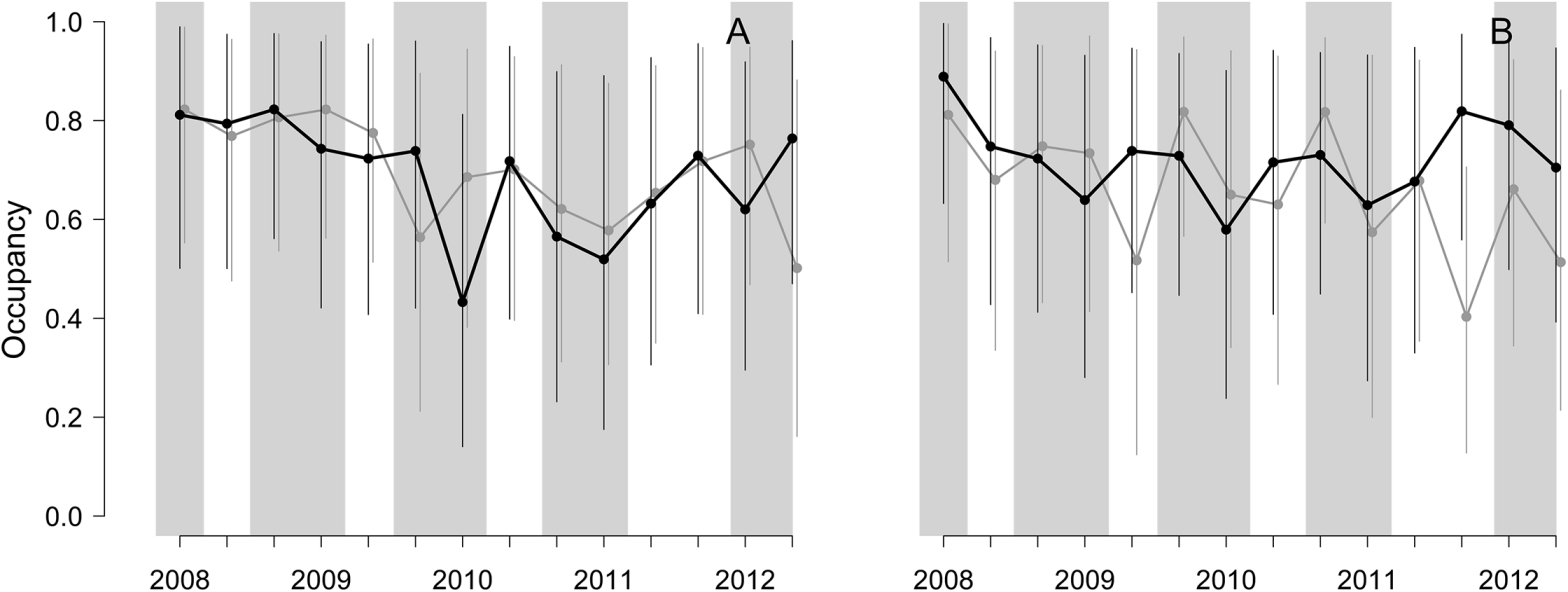
Dynamic site-occupancy for *Martes* spp. (A) and red fox *Vulpes vulpes* (B) during the six-year experiment, in the treatment plot T (in which mesocarnivore removal was performed, black dots-lines) and in the control plot C (with no mesocarnivore removal, grey dots-lines). Occupancy data refer to the first three quarters of the year (January-August) including life-cycle phases such as wintering, mating display, incubation and the chick-rearing period of the capercaillie in the Pyrenees. Grey shading corresponds to removal periods.

Focusing on red fox and *Martes* spp., we detected signs of the presence of these species in all the monthly surveys throughout the study period. We detected the presence of red fox in a total of 1220 stretches (32.5% of the total stretches surveyed; 624 and 595 in C and T, respectively), whereas *Martes* spp. was detected in a total of 1503 stretches (40.0%; 822 and 681 in C and T, respectively). Although showing remarkably credible intervals, site occupancy probabilities seemed to follow similar trajectories for *Martes* spp. and red fox in both C and T plots (Fig 2). Ocuppancy over the trapping season and during the following quarter of the year was lower in T in 10 out of 14 cases for *Martes* spp (71.4%), while for the red fox, occupancy was lower in T in 7 out of 14 cases (50.0%; Fig 2). However, occupancy of all species pooled increased again in the second quarter after trapping in 83% of these cases in both T and C. For the *Martes* spp., site occupancy oscillated between 0.43 and 0.82 in T, and between 0.50 and 0.82 in C; for red fox, occupancy oscillated between 0.58 and 0.90 in T, and between 0.40 and 0.83 in C (Fig 2).

### Effect of mesocarnivore removal on capercaillies

**Fig 3:**
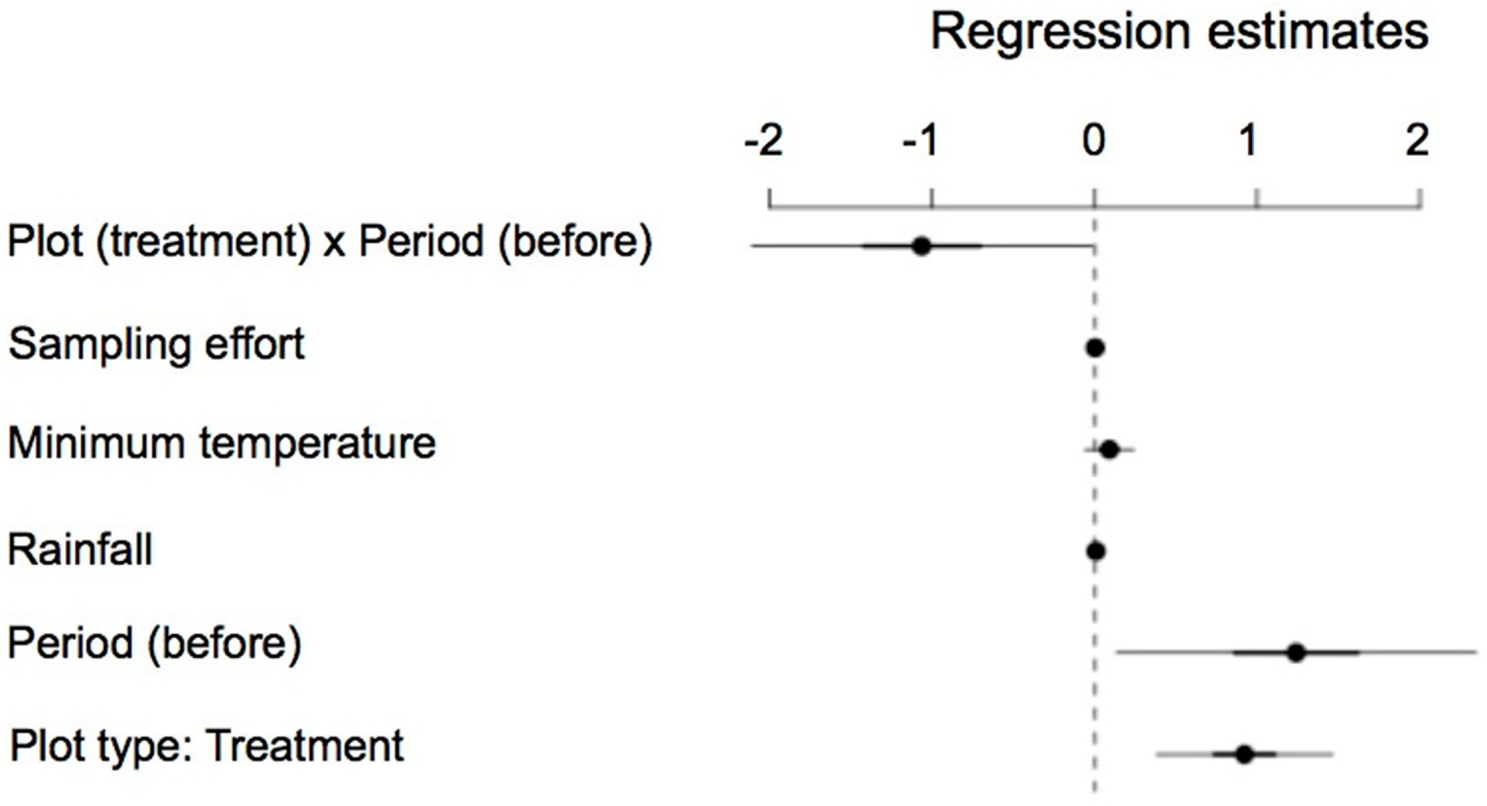
Regression estimates of each of the predictors (in Y-axis) considered in the GLMM analysis of breeding success of Pyrenean capercaillie *Tetrao urogallus aquitanicus*, with the ±50% (thick line) and ±95% (fine line) confidence intervals. *Sampling effort* = area (ha) covered during the census yearly; *Minimum temperature* = minimum °C registered during incubation and chick rearing period (May-June); *Rainfall* = total mm registered during incubation and chick rearing period (May-June); *Period (before)* = phase in which breeding success was considered, before or after the beginning of the removal experiment of mesocarnivores; *Plot type (Treatment)* = type of study plot in relation to the removal or lack of removal of mesocarnivores (treatment = removal). *Plot (treatment) x Period (before) =* interaction of the two former variables following a BACI design.

**Fig 4:**
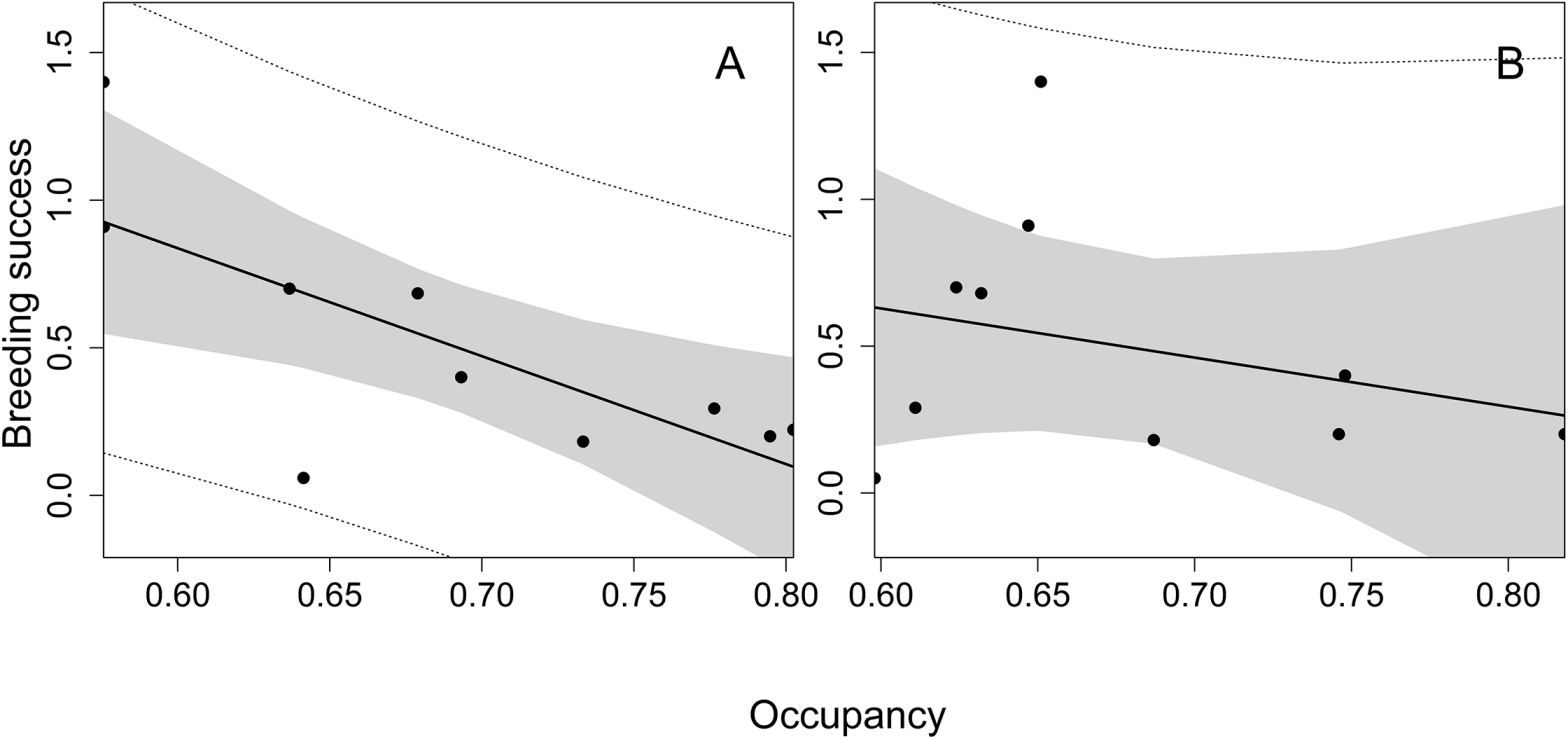
Linear regression (black line) of the values of breeding success (Y-axis) of Pyrenean capercaillie *Tetrao urogallus aquitanicus* and the probability of niche-occupation of *Martes* spp. (A) and red fox *Vulpes vulpes* (B) (X-axis) ±95% confidence interval (shaded area) during the six-year experiment of mesocarnivore removal in both treatment and control plots in the Spanish Pyrenees.

Overall, we recorded individual information on the breeding success for 321 females during the study period (158 cases in T and 163 in C). During the study period, breeding success (i.e. number of fledglings per hen) during the removal experiment (2008–2013) was as twice as high in T compared to C (0.57 *vs*. 0.28, respectively) while these figures values similar before the start of the removal experiment between plots (1999–2007; 0.53 *vs*. 0.56 in T and C, respectively). Differences in breeding success between the treatment and control plots before and after were statistically significant (interaction term plot type x period: *χ* ^*2*^ = 4.00, df = 1, *P* = 0.045) when covariates were controlled for (rainfall, *P* = 0.006; minimum temperature, *P* = 0.238; effort, *P* = 0.390) (Fig 3). The Zero-inflated model approach produced the best fit to the data (lower AIC; Zero-inflated model AIC: 526.5; Negative binomial model AIC: 528.9; Poisson model AIC: 615.1). No overdispersion was observed (1.1). Breeding success was negatively correlated with the occupation rate of *Martes* spp. (r = -0.655; F_1_ = 7.511; *P* = 0.019) (Fig 4). For the red fox, although the same negative trend was observed, it was not statistically significant (r = -0.288; F_1_ = 0.718; *P* = 0.421) (Fig 4).

Regarding adult survival rates based on VHF-marked animals, we monitored capercaillies on average for 471 days (range 32–1526), with the monitoring effort being similar between T and C plots (a mean of 350 and 530 days, respectively; Mann-Withney U-test, *P* = 0.291). Of the 11 radio-tracked capercaillies monitored in T, none died during the study period (annual survival close to 1 although due to the lack of deaths it was not possible to estimate the exact value). However, from the 22 radio-tracked individuals in C, 3 were predated (annual survival = 0.62±0.10). We did not detect statistical differences in cumulative survival between T and C (*χ* ^*2*^ = 1.20, df = 1, *P* = 0.275). Finally, we detected 35 clearly predated non-juvenile capercaillies during the study period, 9 in T (4 *vs*. 5 before and after, respectively) and 26 in C (8 *vs*. 18 before and after, respectively). Eleven out of these 35 events were classified as predation events by mesocarnivores (at least 5 by *Martes* spp. and the rest by unidentified mesocarnivore species), 10 as predation events by raptors and 14 as unknown. The overall predation rate, i.e. number of predation events per 1000ha, was 1.43 in T (1.15 before and 1.79 after) and 2.96 in C (1.97 before and 3.61 after) while the predation rate due to mesocarnivores was 0.63 in T and 1.03 in C. Nevertheless, the probability of finding a capercaillie carcass predated upon by mesocarnivores did not differ between the T and C plots before and after (interaction term period x plot: *χ* ^*2*^ = 0.034, df = 1, *P* = 0.854). The same result was found when we merged predation events by mesocarnivores with unknown cases (*χ* ^*2*^ = 0.256, d.f. = 1, *P* = 0.613).

## Discussion

We observed a reduction in mesocarnivore site occupancy (reference mesocarnivores: *Martes* spp. and red fox) in T during the crucial annual periods for capercaillies of hatching and laying. However, once removals ended, most of the time occupancy for reference mesocarnivores recovered between removal seasons (Fig 2). On the other hand, site occupancy patterns of mesocarnivores were not very dissimilar between C and T (Fig 2), suggesting the presence of external factors influencing occupancy patterns at a regional scale beyond our intervention (e.g. food availaibity, weather conditions). Emptied territories probably acted as sinks admitting new individuals from surrounding areas in the short-term, with removed individuals being replaced by younger dispersing individuals [35,79]. The number of captures declined gradually over time, and this pattern was unrelated to trapping effort, which may indicate a general decrease in the abundance of mesocarnivores in T. There are alternative non-mutually exclusive scenarios that could explain this pattern, but the lack of previous abundance data prevents further elucidation. First, extractions may have produced an effective reduction in mesocarnivore numbers, but the infringement of the closure assumption due to the removal of some individuals between primary periods may overestimate the values of site-occupancy in T, given the high probability of colonization and low probability of extinction in the emptied territories, although this deviation would be low [80]. Second, removal may not have produced any relevant change in red fox populations due to a potential lack of trapping effectiveness. Consequently, remaining individuals may have continued using the same paths, marking them more actively [81].

Our findings show a significant effect of mesocarnivore removal on capercaillie breeding success (number of fledglings per female) [31,33,82]. In fact, breeding success values slightly recovered in T after the start of the removal experiment. Thus the largest brood sizes in the Catalonian Pyrenees in recent times were recorded in T during the experimental period: 6 (n = 1) and 4 (n = 2) chicks/female. Moreover, we found a significant effect of occupancy of *Martes* spp. but not of red fox on breeding success, thus discerning a potential heterogeneous impact on capercaillies from different mesocarnivores. Little evidence is available on the role of predation in adult mortality and survival rates in capercaillies. However, no radio-tagged capercaillies died during the study period, whereas 3 animals died in C because of predation by mesocarnivores. This result supports the idea that, in addition to breeding success, other demographic parameters may also benefit from predation management [11,22,83]. The increase in breeding success and adult survival, as a result of mesocarnivore removal, is seemingly higher in those studies that removed all predator species than those that removed just a subset [21].

Possible limitations to our study should be considered regarding the accuracy of the proxies of breeding success. Censuses are conducted when chicks are developed enough to fly, so it is expected that they will flush with the hen. However, experience with radio-collared females shows that hens sometimes leave their fledglings hidden under ground cover and flush without them, as a form of protection. This behaviour could potentially affect our results. However, due to habitat similarity it is plausible to expect that this effect should be homogenous throughout the study area and between plots, similarly affecting C and T plot estimates over time. Thus, we assume that this potential effect is not responsible for the main differences we observed between plots. On the other hand, we were able to include only one C and T plots in our study design. However, despite it is recommended to perform this kind of analyses replicated, there are also examples of BACI designs using single experimental plots [84]. Furthermore, it is worth mentioning that these plots were larger enough (ca. 1,000 ha) to host a remarkable number of all the studied species, including mesocarnivores with large spatial requirements (home ranges between cs. 150 and 250 ha), as well as the difficulty in finding comparable plots in the study area (regarding vegetation, climate and land-use characteristics) or the availability of previous counts of capercaillies with standardized protocols. We balanced these issues during experiment design and concluded that, given our particular conditions, two 1,000 ha plots was efficient enough to tetst the effects on capercaillie in our Pyrenean study area. Finally, we were focused only on terrestrial mesocarnivores, so the effect of crows and raptors deserves further investigation [33,85]. It is possible that aerial predation may reduce the increase in capercaillie breeding success produced by the removal of terrestrial predation [86].

In light of our results, intuitively, the observed patterns would indicate that mesocarnivore control could be considered as an effective management or conservation tool [25]. The doubled productivity found in the removal area of mesocarnivores would suggest that such an effort maintained over time may allow prey populations to recover if they have been subjected to predator pits [87], or when few specialized individuals account for the bulk of prey mortality [88]. However, this strategy would be very time-consuming as well as logistically and financially constrained (see Table 1). In practice, complete removal would be almost impossible in open areas and in ecosystems where the predator guild is as diverse as it is in our study area. Moreover, some experiences have called into question the effectiveness of lethal management tools and the undesired side-effects of an excessive or insufficient predator control [89,90]. The negative societal views and ethics associated with these practices and the conservation needs of target species (predators and prey), lead to the consideration of alternative management strategies to reduce the impact of mesocarnivores on prey populations, such as capercaillies.

Thus, cost-effective, long-term management interventions are required to ensure the recovery of this threatened capercaillie population at a population scale. A more feasible and sustainable management intervention in ecological, sociological and economic terms, may be to balance the impact of mesocarnivores on capercaillies through the recovery of apex predators, i.e. restoring the predator community with apex predators [12,91,92]. Within the geographical scope of this study, mesocarnivore abundances, especially those responsible for higher predation rates (red fox and mustelids), may be naturally balanced by the presence of apex predators, reversing the impacts of mesopredator release process [1,7]. In our case, evidence suggests that restoring the predator community of this alpine area by bringing back the Eurasian lynx which was present in mountain areas of northen Spain until the twenteenth century [93,94], might be beneficial for capercaillies through triggering mesocarnivore cascading effects [3,5,6,95]. We believe this species provides a much better restoring effect on the entire carnivore community due to common intraguild predation to other mesocarnivores (red fox and martens) in comparison to other apex predators disappeared or very scarce in the study area, as the grey wolf and the brown bear [96,97]. For example, the recovery observed of Eurasian lynx in recent times in Finland [98] was accompained by a decline in red fox abundance as well as a recovery in the abundance of forest grouses (including capercaillies) [5,6]. Apart from direct effects, non-lethal, behaviourally-mediated effects of Eurasian lynx on mesocarnivores would be expected, such as changes in habitat use or foraging patterns (i.e., landscape of fear) [99,100]. Moreover, this apex predator could also have a medium-long term positive effect due to its potential control effect of wild ungulates [2,4,6], whose overabundance in the Spanish Pyrenees is another factor related to capercaillie decline [30]. Finally, as a conclusion to the adaptive management procedure applied in our study (establishment of a clear purpose and model design, development of a monitoring and management program, data analysis and communication of results) [36], we recommend a scientific assessment of the potential impacts of restoring the Eurasian lynx on not only capercaillies, but also on the entire alpine ecosystem in the Pyrenees.

## Supporting Information

S1 Fig Pyrenean female capercaillie *Tetrao urogallus aquitanicus* radiotagged. (DOCX)

S2 Fig Capercaillies *Tetrao urogallus aquitanicus* found predated by mesocarnivores during the study period. These two examples show a radiotagged adult breeder female recently predated in the control area, by *Martes* spp. in August 2010 (see faeces of *Martes* spp. next to the corpse, in A) and by a raptor (possibly goshawk *Accipiter gentilis*) in June 2009 (B).
(DOCX)

S1 File Data underlying the findings described in this manuscript. (ZIP)
