# Correction: Is It Necessary Managing Carnivores to Reverse the Decline of Endangered Prey Species? Insights from a Removal Experiment of Mesocarnivores to Benefit Demographic Parameters of the Pyrenean Capercaillie

**DOI:** 10.1371/journal.pone.0216909

**Published:** 2019-05-13

**Authors:** Rubén Moreno-Opo, Iván Afonso, José Jiménez, Mariana Fernández-Olalla, Jordi Canut, Diego García-Ferré, Josep Piqué, Francisco García, Job Roig, Jaime Muñoz-Igualada, Luis Mariano González, José Vicente López-Bao, the PLOS ONE Editors

After publication of this article [1], questions were raised about the reporting of the mortality associated with the methods used for bird capture. Here, the authors provide additional information regarding the methods used and mortality observed in the study to clarify this issue.

Additionally, during the journal’s follow-up on the concerns raised in this case, the *PLOS ONE* Editors became aware of a potential competing interest between the original handling Academic Editor and some of the co-authors of the article that was not appreciated during the review process. In light of this, we had the article reassessed by another member of our Editorial Board who confirmed that the article, with the following amendments, meets *PLOS ONE*’s publication criteria.

Please see the additional information provided by the authors here:

## Regarding live-bait cage traps

As per Spanish legislation, capture of wildlife is permitted for the purpose of research provided there is no alternative means of addressing the proposed study aim, and researchers must adopt a capture and tagging method that minimizes the risk of injury or mortality to the captured individuals (article 61.7 of Law 42/2007). For this study [1] we opted for the use of live-bait cage traps in seasons 1–4 (of 6 total) due to their effectiveness for species such as the wildcat *Felis silvestris* and common genet *Genetta genetta* [2] in relation to other trapping systems with non-live baits. All the approved trapping devices had systems to avoid negative effects on the welfare of the animals during the capture process: domestic pigeons *Columba livia* used as lure came from captive breeding farms, were placed in boxes with food, water and shelter in the face of inclement weather, and were housed in pairs during the capture period, in cages attached to trap-boxes where they were not accessible to predators. No bait animals suffered damage or depredations during the study. Once we concluded the work with these devices, domestic pigeons were returned to the breeding farms where they came from. As we focused fox *Vulpes vulpes* and martens *Martes martes*, *Martes foina* as the target monitored species from the third year onwards, the use of live-baited cage traps was not needed due to the existence of other more selective and efficient systems (non-live baited cage traps and Collarum) for these predators that did not require the use of live animals as decoys. All captured carnivores were translocated to approximately 100 km from treatment plots, with the exception of red foxes, which were legally euthanized (n = 12) in compliance with its legal status as a hunting species in Spain. To check for potential homing effects [50], which would invalidate our experiment, we marked a random sample of every translocated species (about one in every three captures sequentially) using subcutaneous tags (Freevision, 0.15 g, ISO 11784/785), to determine if they were re-captured after translocations in the treatment plots. We used a simple 2 x 2 BACI (before-after-control-impact) experimental design, in which parameters of interest were measured before and after treatment, for both control and treatment conditions, because of the logistical constraints of this type of large-scale experiment and the spatial behaviour of target species (plots of ca. 1,000 ha and mesocarnivores with home ranges of several hundreds of ha).

## Capercaille monitoring and mortality

In relation to the mortality rates of capercaillie *Tetrao urogallus* and its relationship with the capture techniques implemented, there are several issues that require a detailed explanation with the purpose of distinguishing different parameters. First of all, the monitoring of radiotracked birds in our project (2008–2013), which is the subject of analysis in the article, showed a mortality of 22.9% (11 of 48 studied totals) of the tagged birds. But these birds did not die as a result of tagging techniques that could cause injuries or stress from capture and handling, or conditioning of the behaviour by the presence of transmitter. These were animals that died as a consequence of natural causes, all of them due to natural depredation (from carnivores or raptors) after a variable number of days of monitoring after the capture and radioequipment: 38 days (n = 1), 43 (n = 1), 73 (n = 1), 95 (n = 1), 248 (n = 1)), 380 (n = 1), 403 (n = 1), 469 (n = 2), 690 (n = 1), 922 (n = 1). From these 11 deaths, six were female and five were male. It is important to note that in this analysis only the capercaillies that exceeded a period of presence of the transmitter exceeding 32 days were included. Therefore, it should be stressed that this mortality is not attributable in any case to the type of tagging done nor to the management conferred on birds, but strictly to natural causes such as predation. That was, precisely, the object of study in our case (to assess the survival rates of birds and the causes of mortality in different areas). This information can be seen on the sheet called "original" of the file included with the name "copia mortalitat_8gen14" of the supplementary information (see S1 File of [1]).

Notwithstanding, more data were obtained from tagged birds that died before exceeding the 32-day duration of the transmitter and that are also shown on the sheet called "mortalitat" in the file included with the name "copia mortalitat_8gen14" of the supplementary information (see S1 File of [1]). These birds were not included in the survival study because they did not exceed the minimum number of days of study necessary to be able to evaluate their survival comparatively. In this case, some deaths came as a result of the tagging activity, so we were able to verify under what circumstances the capture of this species has to be avoided. In addition to the 48 birds for which survival was analyzed, another 13 birds were captured during our project (2008–2013). In those 13 birds the transmitters operated for a maximum of 32 days. The causes of suspension of monitoring of these birds were the following:

- 7 birds (5 females and 2 males) died. Of the 7 birds found dead, 2 died as a consequence of capture stress (myopathies) based on a necropsy. The other 5 birds found dead had clear evidence of predation: however, it is not completely sure that both the predation and possible scavenging could have been produced after the death of the animal, perhaps due to the stress of capture. The necropsies performed on these 5 animals could not determine if there were physical effects derived from the handling of the bird. In several of these individuals only feathers and few other tissues were found.
- 6 birds lost the transmitter due to its poor fitting on the bird (the fallen transmitters were found without any trace of mortality of these birds).

Based on the above, a sure percentage of mortality due to capture effect can be established at 3.2% of the total 61 (48 + 13) birds captured during the project. In relation to the rest of birds that died before 32 days and that showed signs of predation, we cannot say if predation arose due to the poor physical condition of the bird as a consequence of the capture. Therefore, if these 5 birds are summed to the other 2, it could be speculated that the mortality caused by the capture of the bird could have reached a maximum of 11.4% (7 of 61 birds).

As a consequence, during the project we observed some lessons that modulated the capture procedures for radiotracking capercaillies:

- The females captured during the exhibition and mating period in the leks (May) were more susceptible to being affected by the stress of capture and all the birds that died by myopathy had been captured during this period. Therefore, in 2012 the capture of females was suspended during the exhibition and mating period. On the other hand, males did not have a higher mortality rate due to being captured during exhibition time.
- In contrast to the mating/exhibition period, during the chick-rearing period (months from July to September), as well as after this time (October to November), the females were not affected by the stress of capture in any of the cases.
- The techniques of handling and tagging were agile and fast, not keeping the animal retained more than 2 minutes in any of the cases before its release with the transmitter placed.
- Sedation techniques were not used during the project. After the end of the study and with the purpose of improving handling and tagging procedures, sedation trials were tested both with domestic chickens *Gallus gallus domesticus*, as with other species (ptarmigans *Lagopus muta*, hazel grouse *Bonasa bonasia*) and with the capercaillies themselves between 2017 and 2018 in France and Spain.
- Despite the undesirable loss due to mortality of some capercaillies during the capture and radiotagging tasks, valuable information could be obtained about the best protocols for an activity that had never been tried before, or at least communicated by experts or shown in reference publications. In this sense, the best techniques to tag capercaillie females have been publicized by our team to a wide audience that works with this and other similar species in recent years.

From the analyzed and monitored individuals, 22.9% died from natural causes; that is, they would have died as well without being radiotagged. The proven mortality rate caused by the capture, handling and/or radiotagging effect was 3.2%.

The capture, management and transmitter-setting techniques were authorized by the relevant administrations (Generalitat de Catalunya and Ministry of Environment) following the legislation applicable in Spain in relation to research purposes in wild animals (Article 61 of Law 42/2007, and Article 9 of the Birds Directive 147/2009/EC). The issued permits established the working protocols, methods and types of activities allowed. In this sense, the Spanish legislation itself indicates the need for applying best practices for capture and tagging which must adopt the alternative with less probability of causing injuries or mortality of the captured individuals (article 61.7 of Law 42/2007). All the conditions stipulated by the environmental authorities were followed during the fieldwork of capture and marking of capercaillies in our study, strictly following the regulations regarding animal protection and welfare applicable in Spain.

In relation to compliance with IACUC standards, our study did not follow its adoption as it was not legally applicable in Spain, we did not work with laboratory animals and because there are other recommendations/guidelines to be fulfilled: The technical standards for ringing birds, the compulsory communication of the activities to the European Commission–HABIDES database- and the justification report sent to the competent authority -the Generalitat de Catalunya-.

## References

[1] Moreno-Opo R, Afonso I, Jiménez J, Fernández-Olalla M, Canut J, García-Ferré D, et al (2015) Is It Necessary Managing Carnivores to Reverse the Decline of Endangered Prey Species? Insights from a Removal Experiment of Mesocarnivores to Benefit Demographic Parameters of the Pyrenean Capercaillie. PLoS ONE 10(10): e0139837 10.1371/journal.pone.0139837 26489094 PMC4619448

[2] Barea-Azcón JM, Virgós E, Ballesteros-Duperón E, Moleón M, Chirosa M. Surveying carnivores at large spatial scales: a comparison of four broad-applied methods. Biodiv. Conserv. 2007;16: 1213–1230. 10.1007/978-1-4020-6320-6_26

