# Supplementary material for: Is It Necessary Managing Carnivores to Reverse the Decline of Endangered Prey Species? Insights from a Removal Experiment of Mesocarnivores to Benefit Demographic Parameters of the Pyrenean Capercaillie
Source: PLoS One. 2015 Oct 21;10(10):e0139837. doi: 10.1371/journal.pone.0139837 (PMC4619448; doi:10.1371/journal.pone.0139837)

**Supporting Information**

**S1 Fig.** Pyrenean female capercaillie *Tetrao urogallus aquitanicus* radiotagged.


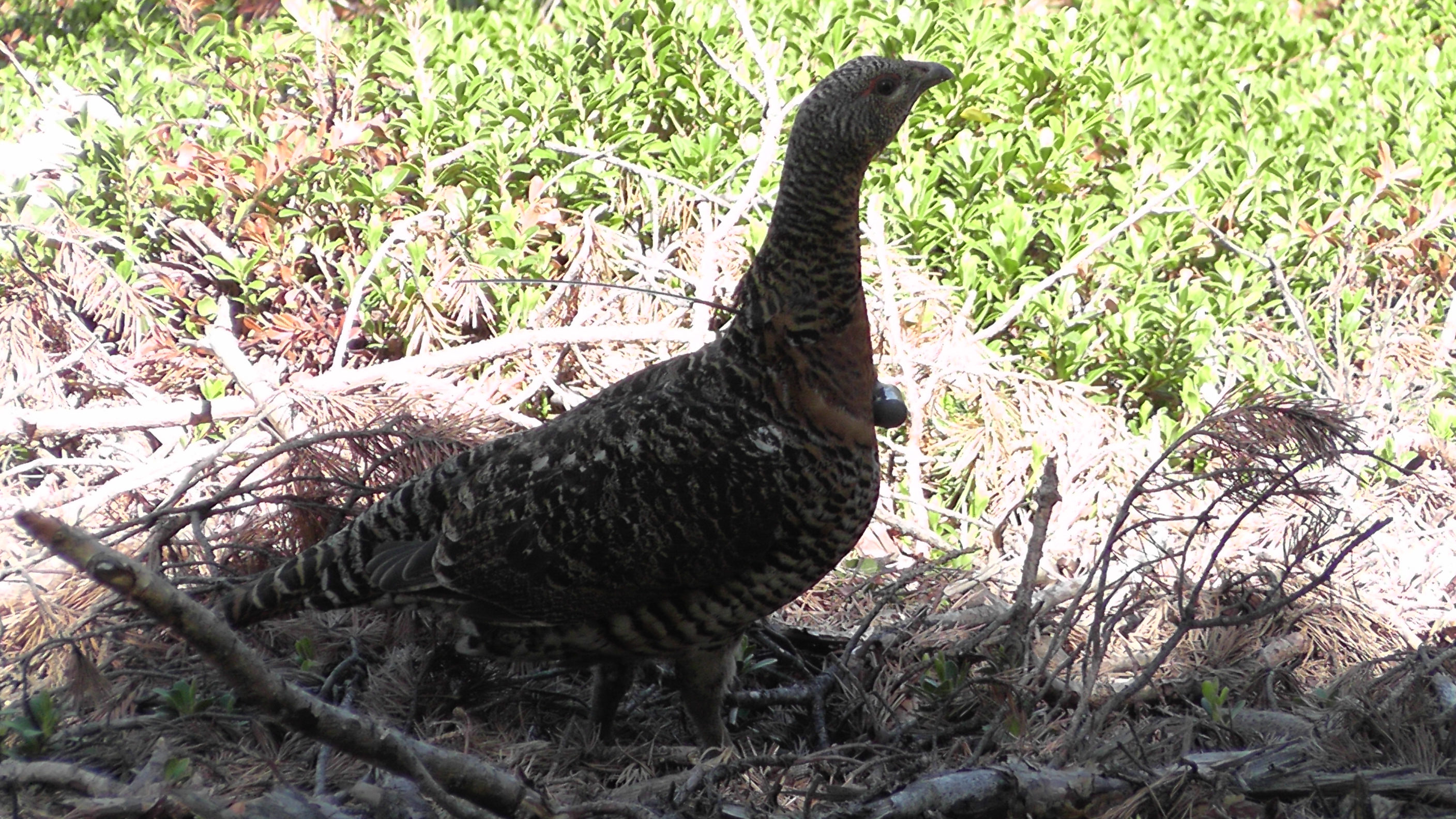


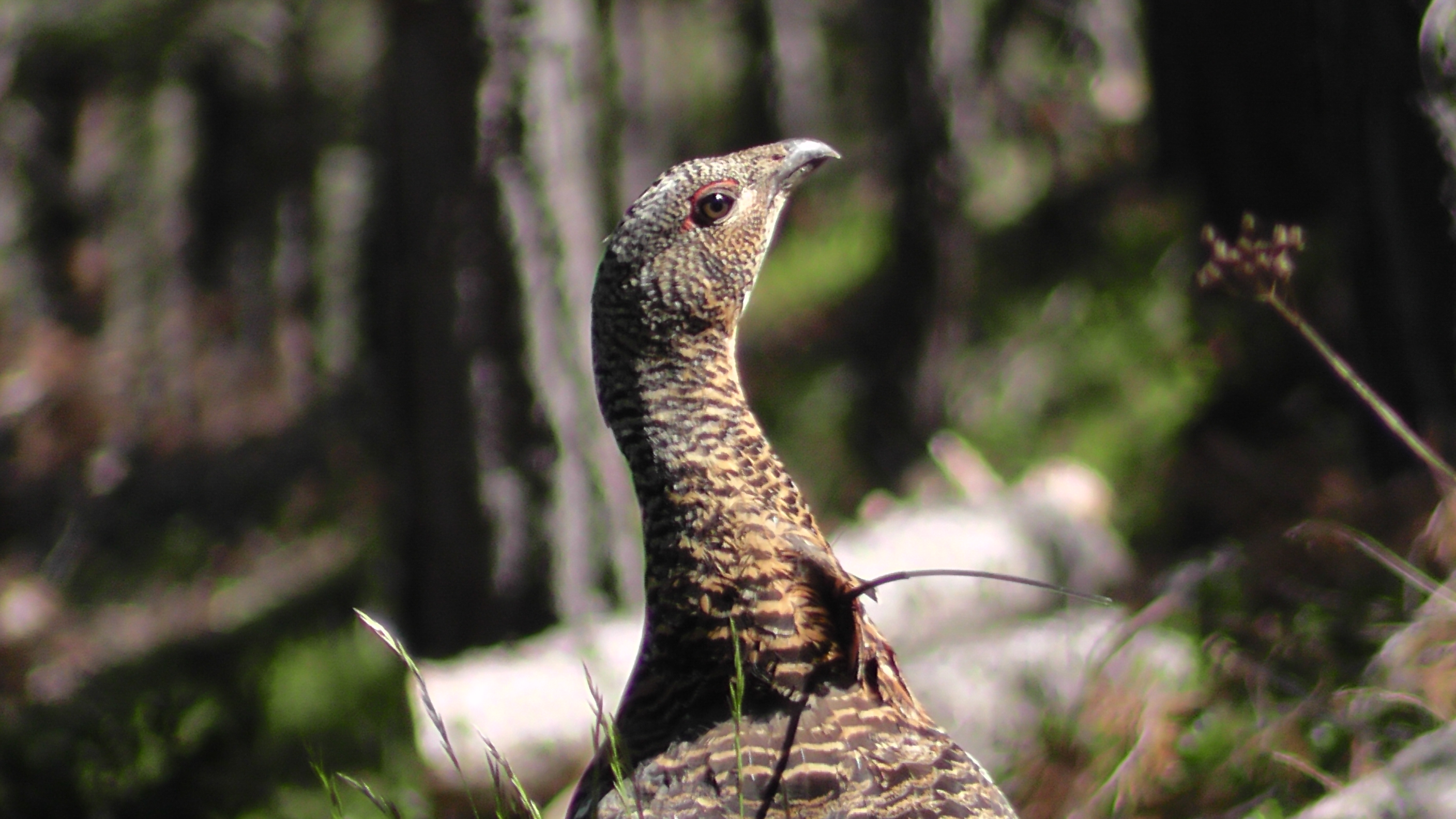

Supplement: S1 Fig — (DOCX) [file pone.0139837.s001.docx]
