# Supplementary material for: Is It Necessary Managing Carnivores to Reverse the Decline of Endangered Prey Species? Insights from a Removal Experiment of Mesocarnivores to Benefit Demographic Parameters of the Pyrenean Capercaillie
Source: PLoS One. 2015 Oct 21;10(10):e0139837. doi: 10.1371/journal.pone.0139837 (PMC4619448; doi:10.1371/journal.pone.0139837)

**Supporting Information**

**S2 Fig.** Capercaillies *Tetrao urogallus aquitanicus* found predated by mesocarnivores during the study period. These two examples show a radiotagged adult breeder female recently predated in the control area, by *Martes* spp. in August 2010 (see faeces of *Martes* spp. next to the corpse, in A) and by a raptor (possibly goshawk *Accipiter gentilis*) in June 2009 (B).

.

A
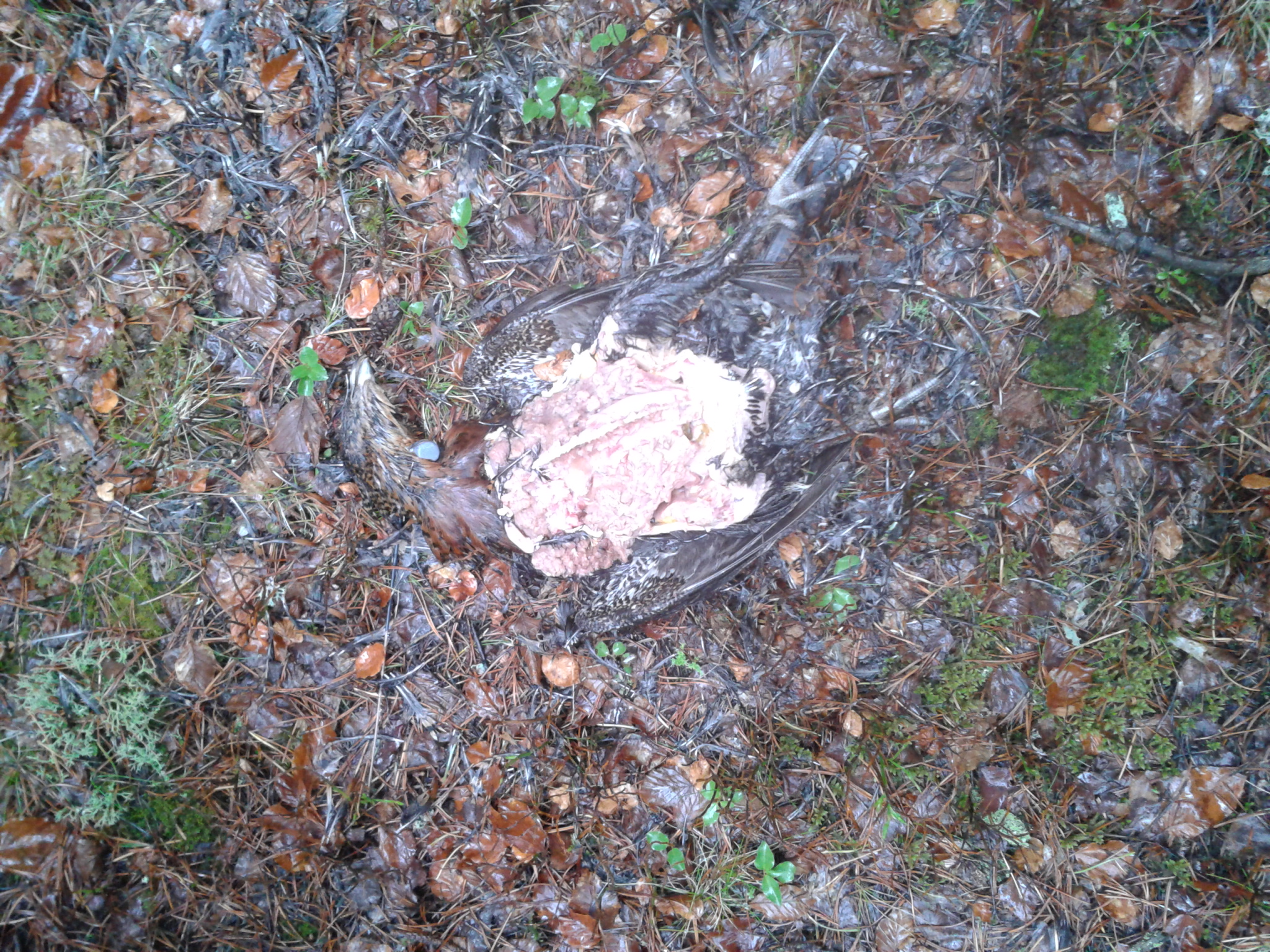


B
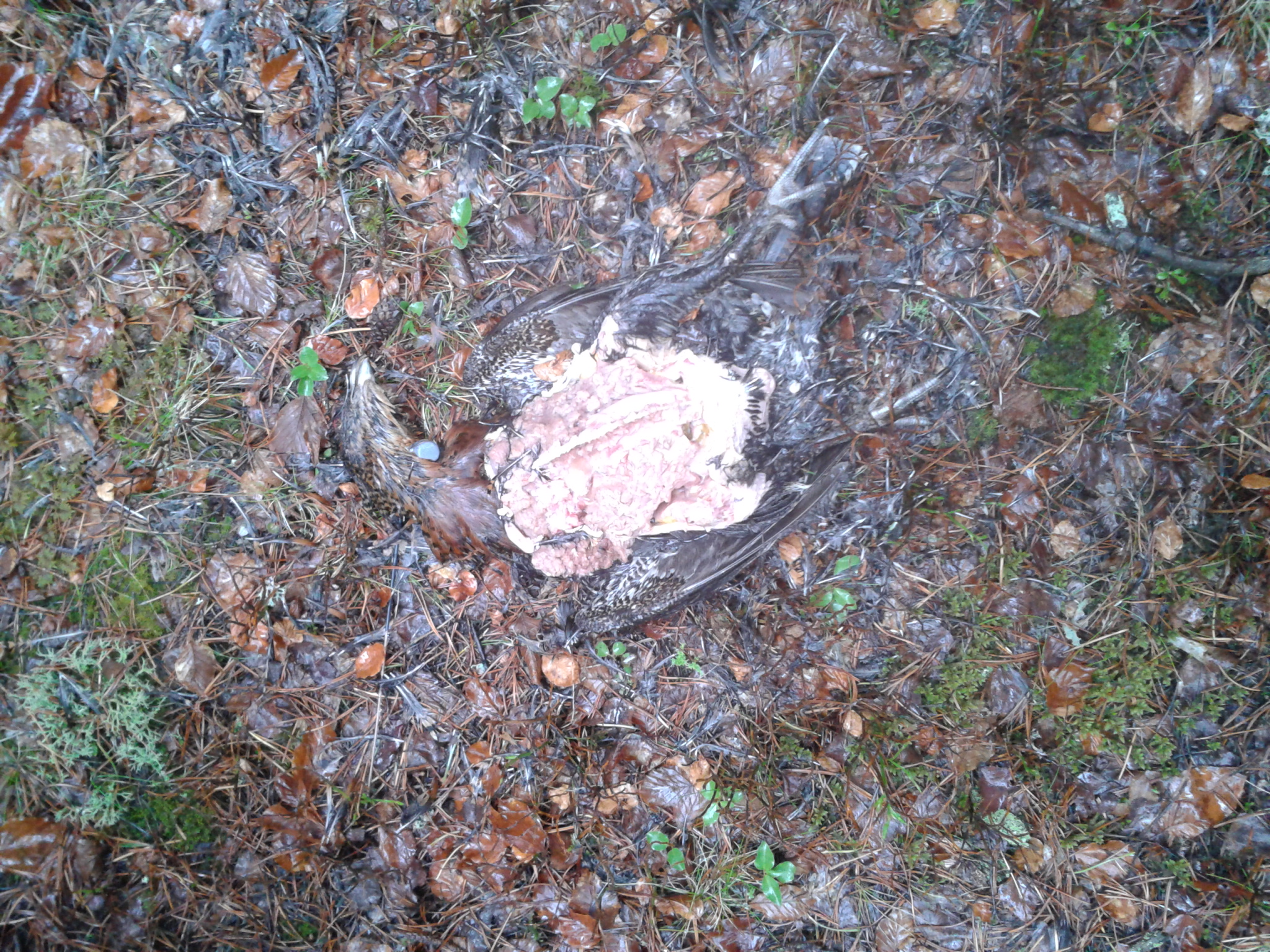


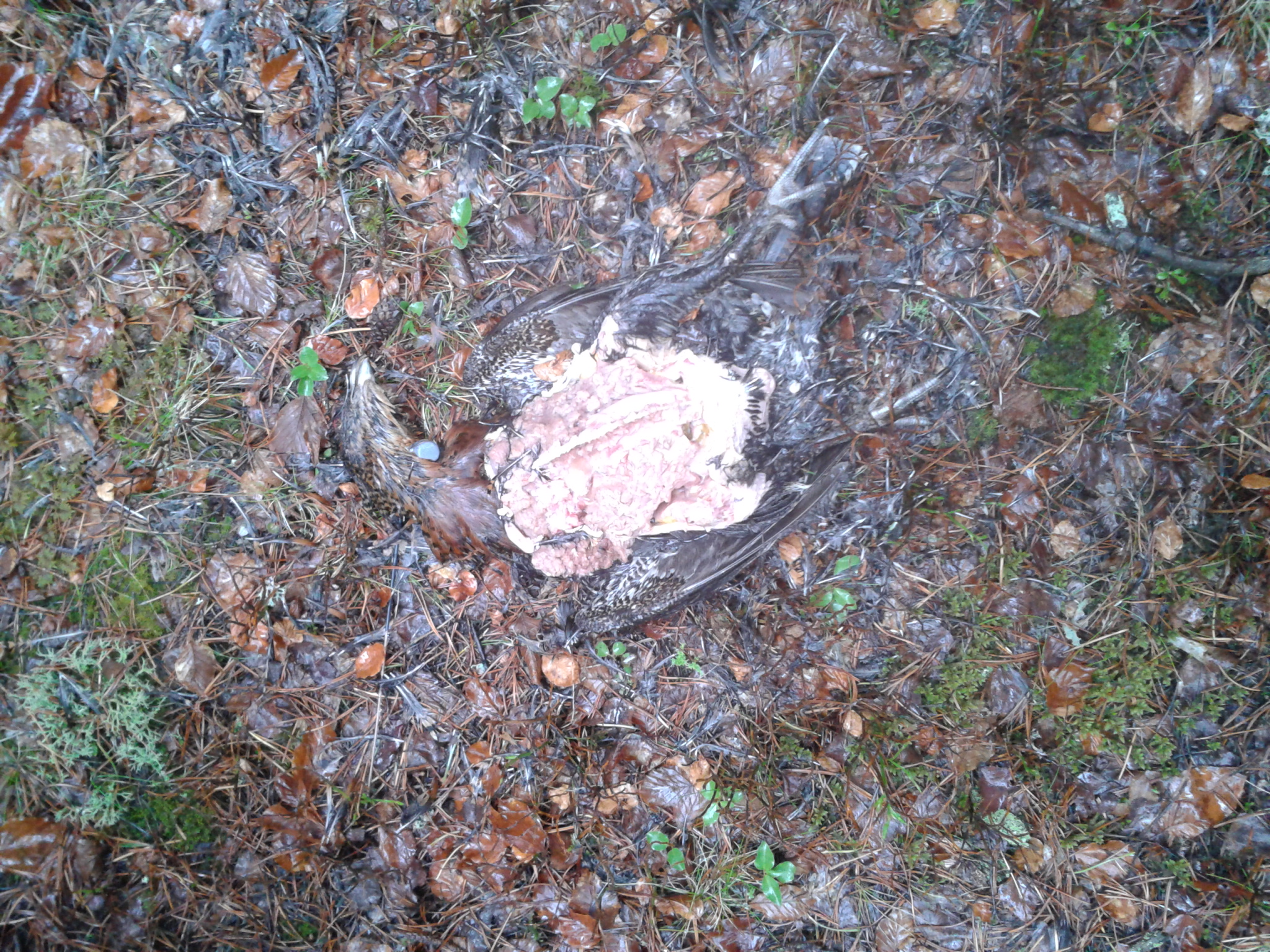


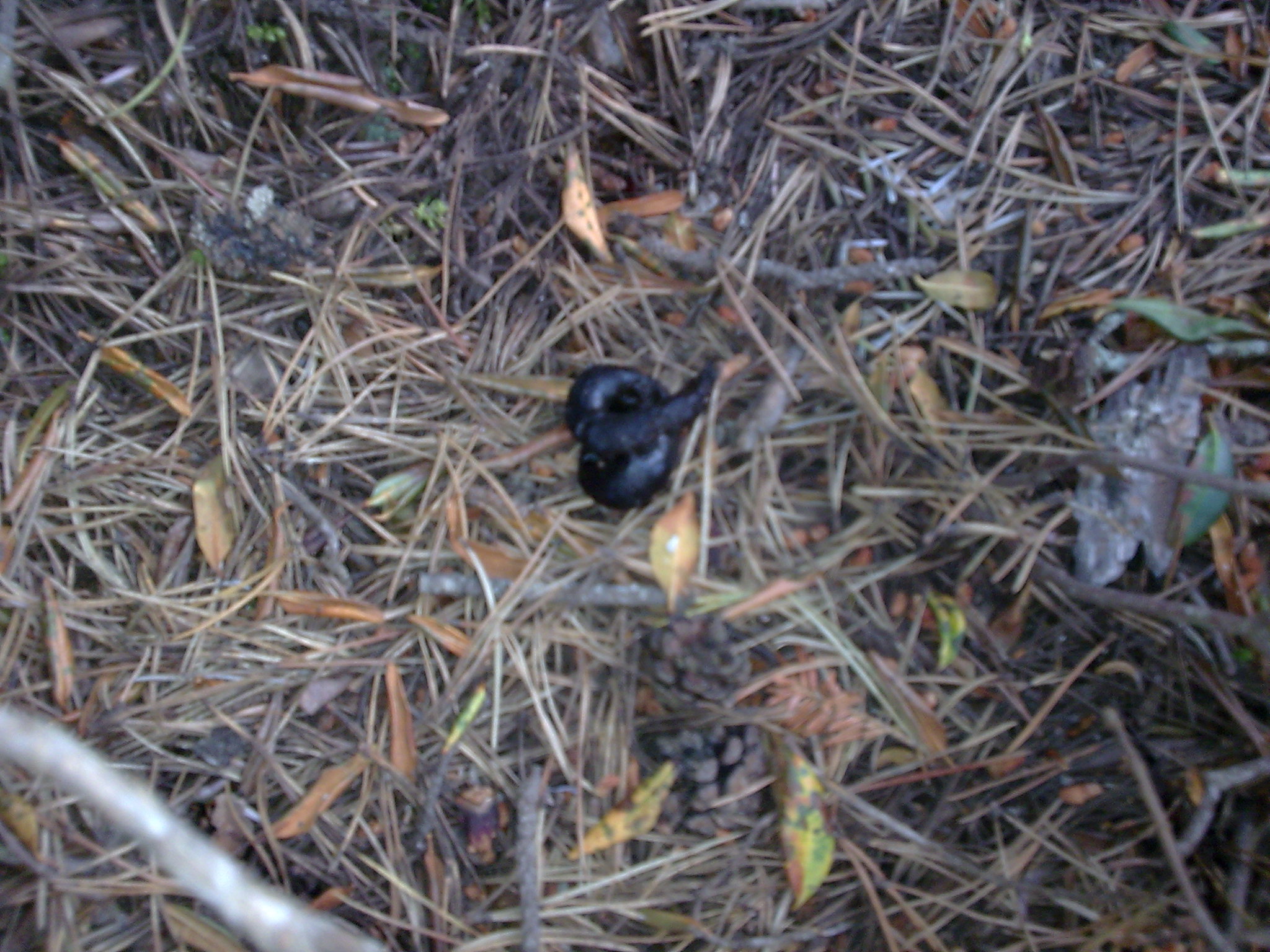


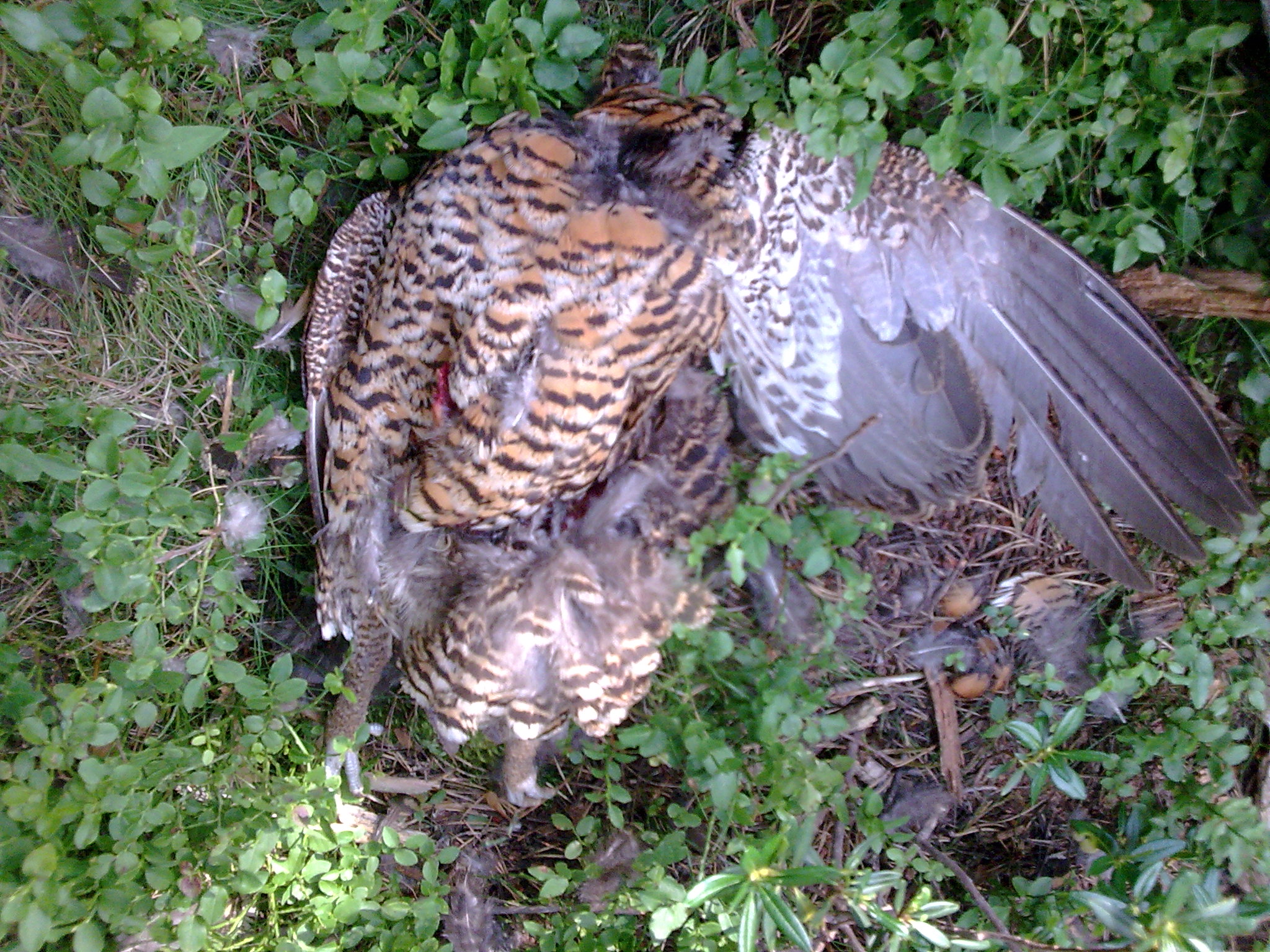

Supplement: S2 Fig — These two examples show a radiotagged adult breeder female recently predated in the control area, by Martes spp. in August 2010 (see faeces of Martes spp. next to the corpse, in A) and by a raptor (possibly goshawk Accipiter gentilis) in June 2009 (B). (DOCX) [file pone.0139837.s002.docx]
